# Hypoxia, HIF, and Associated Signaling Networks in Chronic Kidney Disease

**DOI:** 10.3390/ijms18050950

**Published:** 2017-04-30

**Authors:** Jing Liu, Qingqing Wei, Chunyuan Guo, Guie Dong, Yu Liu, Chengyuan Tang, Zheng Dong

**Affiliations:** 1 Department of Nephrology, The Second Xiangya Hospital, Central South University, Changsha 410011, China; jinliu@augusta.edu (J.L.); rory0423@163.com (Y.L.); tangchengyuan@hotmail.com (C.T.); 2 Department of Cellular Biology & Anatomy, Medical College of Georgia at Augusta University and Charlie Norwood VA Medical Center, Augusta, GA 30912, USA; qwei@augusta.edu (Q.W.); chguo@augusta.edu (C.G.); gdong@augusta.edu (G.D.)

**Keywords:** hypoxia, HIF, CKD, fibrosis

## Abstract

The pathogenesis of chronic kidney disease (CKD) is complex and apparently multifactorial. Hypoxia or decrease in oxygen supply in kidney tissues has been implicated in CKD. Hypoxia inducible factors (HIF) are a small family of transcription factors that are mainly responsive to hypoxia and mediate hypoxic response. HIF plays a critical role in renal fibrosis during CKD through the modulation of gene transcription, crosstalk with multiple signaling pathways, epithelial-mesenchymal transition, and epigenetic regulation. Moreover, HIF also contributes to the development of various pathological conditions associated with CKD, such as anemia, inflammation, aberrant angiogenesis, and vascular calcification. Treatments targeting HIF and related signaling pathways for CKD therapy are being developed with promising clinical benefits, especially for anemia. This review presents an updated analysis of hypoxia response, HIF, and their associated signaling network involved in the pathogenesis of CKD.

## 1. Introduction

Chronic kidney disease (CKD) is a condition characterized by the gradual loss of kidney function over time. In the USA, 26 million adults have CKD and millions more are at increasing risk of CKD according to the data of National kidney Foundation. In China, CKD affects approximately 10.8% of the total adult population [1]. The large CKD population imposes huge economic and health burden on both the affected families and the whole society.

Diabetes, hypertension and glomerulonephritis are the leading causes of CKD and multiple underlying mechanisms have been suggested. Among them, hypoxia response and HIF have been noted to play critical roles [2,3,4]. Hypoxia response is one of the mechanisms for kidneys to adapt to the oxygen deficient condition and to survive under pathological conditions. Adequate oxygen is essential for all mammalian organs to fuel various bio-metabolic processes and to maintain biological homeostasis. The balance between oxygen consumption and supply is critical especially for kidneys, which always stay in active metabolic condition and are in high need of oxygen supply. Hypoxia, insufficient supply of oxygen, has been considered to be closely related to CKD progression. The induction of hypoxia during CKD is indeed multifactorial, including increased oxygen consumption, malfunction of microvasculature, vascular remodeling, anemia associated impaired oxygen delivery, impaired oxygen diffusion by extracellular matrix (ECM) accumulation, and mitochondrial abnormality [5,6,7,8,9,10].

The family of hypoxia inducible factors (HIF) plays a central role in hypoxia response. The induction of HIF during hypoxia contributes to renal homeostasis as well as pathophysiology in kidney diseases. Emerging evidence has implicated HIF and hypoxia response in various types of CKD [2,3,4].

In this review, we will mainly focus on the latest research advances with regard to the regulation and role of HIF and hypoxia response in CKD, especially their role in the pathological development of renal fibrosis. Moreover, we will also summarize the recent therapeutic studies of CKD with potential approaches by targeting HIF.

## 2. Regulation of Hypoxia Inducible Factors (HIF)

**Figure 1:**
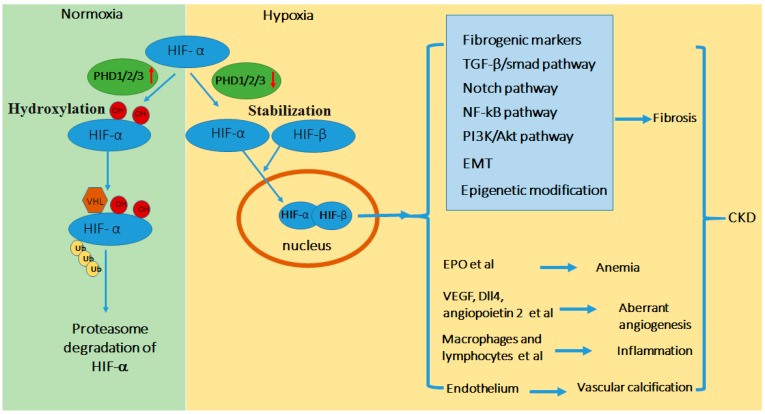
Schematic diagram of HIF regulation in CKD. In normoxia or the presence of O_2_, HIF-α is hydroxylated by prolyl hydroxylase domain (PHD) resulting in its binding of von Hippel Lindau protein (pVHL)-E3-ubiquitin ligase complex, poly-ubiquitination, and consequent proteosomal degradation. In hypoxia or the absence of O_2_, PHD-mediated hydroxylation is inhibited, leading to HIF-α stabilization and accumulation. HIF-α dimerizes with HIF-β to form functional HIF that translocates to nucleus to activate down-stream gene transcription. Hypoxia is a feature in kidney tissues in CKD that activates HIF, which integrates multiple signaling networks to induce renal fibrosis. In addition, tissue hypoxia and HIF contribute to other CKD-associated pathogenic processes including anemia, angiogenesis, inflammation, and vascular calcification. Abbreviations: Epithelial-Meshenchymal Transition (EMT), Erythropoietin (EPO), vascular endothelial growth factor (VEGF), transforming growth factor (TGF).

HIFs are heterodimeric helix–loop–helix transcriptional regulatory factors consisting of a labile subunit HIF-α and a constitutively expressed β-subunit (HIF-β) (Figure 1). The cellular level of HIF-α is regulated by oxygen-dependent proteasomal degradation. Under normoxia, HIF-α is hydroxylated by specific prolyl hydroxylase domain-containing protein (PHD1/2/3), which promotes HIF-α binding to the von Hippel Lindau protein (pVHL)-E3-ubiquitin ligase complex, leading to its polyubiquitination and consequent rapid degradation by proteasome [11,12,13,14]. In addition, hydroxylation of an asparaginyl residue of HIF-α by an asparagine hydroxylase (Factor Inhibiting HIF-1, FIH1) can prevent HIF-α transactivation by inhibiting the recruitment of CBP/p300 that mediates the transcriptional regulation of HIF [15,16,17]. In acute hypoxic conditions, the lack of oxygen supply blocks PHD activity and inhibits the hydroxylation of HIF-α to stabilize it. Consequently, the stabilized HIF-α can dimerize with HIF-β. The dimer then translocates to the nucleus and trans-activates target genes. HIF with its target genes has been proved to function in a variety of biological and pathological process, ranging from fibrosis, angiogenesis, cell proliferation, erythropoiesis, to metabolic switch, inflammation, and apoptosis [11,12,13,14,18,19,20,21,22,23,24]. When hypoxia persists, HIF signaling leads to adaptive responses in order to reduce oxygen demand and increase oxygen supply aiming at reaching a new balance [25].

HIF has three subtypes due to the three isoforms of α-subunits (HIF-1α, HIF-2α and HIF-3α). The major subtypes that mediate hypoxic gene transactivation are HIF-1 and HIF-2. However, they may not be redundant to each other and can play distinct pathophysiological. HIF-1α and HIF-2α share 48% sequence identity and are structurally similar [26]. However, their expression patterns are different which may result in divergent target gene regulation. HIF-1α is expressed ubiquitously in organs in most cell types, whereas the expression of HIF-2α is tissue limited and is particularly detected in highly vascularized organs and tissues [27,28]. In general, HIF-1α governs the initial adaptation process to hypoxia, whereas HIF-2α expression begins after prolonged hypoxic conditions [29,30]. In the kidney, HIF-1α is expressed in most of the cell types with the majority expression in tubular cells, including proximal tubules, distal tubules, connecting tubules, and collecting ducts. Its function ranges from regulating inflammation, fibrosis, apoptosis to glycolysis in hypoxic kidney diseases. However, HIF-2α is expressed mainly in interstitial fibroblasts and peritubular endothelial cells and plays a dominant role in the regulation and induction of Erythropoietin (EPO) production [31,32,33]. The structure of HIF-3α is distinct from both of HIF-1α and HIF-2α with three splicing isoforms [26]. Much less is known about the function of HIF-3. Studies in other research fields show that HIF-3α can act as a target gene of HIF-1 and may negatively regulate the activity of both HIF-1α and HIF-2α [34,35,36]. Some HIF-3 isoforms have transcriptional functions that partially overlap with that of HIF-1α [37,38]. Due to the lack of information about HIF-3 in kidney, in the current article we mainly focus on the regulation and function of HIF-1 and HIF-2 in CKDs.

## 3. HIF in Chronic Kidney Disease (CKD)-Associated Renal Fibrosis 

Renal fibrosis is a pathological hallmark of CKD. Chronic hypoxia has long been considered to be the final common pathological condition for various types of CKDs. Accumulating evidence show that HIF, especially HIF-1α, is a key regulator of renal fibrosis under various pathological conditions [39,40,41,42,43,44,45,46,47,48,49,50,51,52]. However, it is still controversial whether HIF is pro-fibrotic or anti-fibrotic. To define the role of HIF activation on ischemic acute kidney injury (AKI) associated fibrosis, Kapitsinou P et al. inhibited PHD by pharmacological inhibitor to increase HIF before injury, which finally ameliorated AKI-induced fibrosis in mice. However, inhibition of PHD in the early recovery phase of AKI did not show beneficial effects [40]. One possible explanation is that even though HIF activation may reduce renal cell death in ischemic AKI, HIF-1 may actually be pro-fibrotic during recovery phase [40,43,45,46]. In support of this, Wang Z et al. showed that inhibition HIF-1α by short hairpin RNA(shRNA) can attenuate the induction of collagen and α-smooth muscle actin expression in renal artery clamped kidneys [43]. Furthermore, as shown by Higgins DF et al., HIF-1 knockout out in renal epithelial cells can prevent transforming growth factor (TGF) β1-induced epithelial-to-mesenchymal transition (EMT) in vitro and renal fibrosis in vivo [44,45]. Consistently, inhibition of HIF-1α by shRNA attenuates Ang II-induced profibrotic effects [44]. However, in a rat remnant kidney model by removal two-thirds of the left kidney to induce renal fibrosis, treatment with L-mimosiney to activate HIF-1α can attenuate renal tubulointerstitial fibrosis [41]. Furthermore, Kobayashi H et al. showed that global activation of HIF repressed fibrogenesis in mice subjected to unilateral ureteral obstruction (UUO) [42]. Currently, the cause of this significant discrepancy between these studies regarding role of HIF-1 in renal fibrosis is not clear. Although it is generally believed that the inconsistent results from different laboratories can be caused by the variation of experimental conditions which may lead to either protective or detrimental effect, the non-specific effect from pharmacolological inhibitors or shRNAs should also be taken into consideration.

Notably, HIF may play different roles in different renal cells. The pharmacological inhibitors will generally affect all cell types in kidneys, while the inhibition of HIF by genetic methods may only affect specific renal cell types which may contribute distinctly during renal fibrogenesis. In this regard, Pritchett TL et al., showed that conditional knockout of VHL in mouse collecting ducts and a subset of distal tubules results in severe renal fibrosis even without any injury [22]. Notably, the pathological changes in these mice can be rescued by HIF-1α knockout [22]. Similarly, Kimura K et al. showed that VHL deletion causes the accumulation of HIF in tubular epithelial cells that promotes the development of interstitial fibrosis in the 5/6 renal ablation model [46]. In addition, Higgins DF et al. showed that genetic ablation of HIF-1α from renal epithelial cells in mice can attenuate the progression of tubulointerstitial fibrosis in UUO kidneys [45]. In human CKD, there is an association of renal HIF-1α expression and tubulointerstitial injury [45]. Consistently, elevated epithelial HIF-1α levels exacerbate the progression of kidney damage and renal fibrosis in a rat model of hypertension induced by high-salt diet and nitric oxide withdrawal [47]. Altogether, this evidence implies that activation of HIF-1α signaling in renal epithelial cells may accelerate fibrogenesis in CKD.

Several studies have also examined the roles of HIF-1 and HIF-2 in the glomerular endothelium in CKD. Luo R et al. suggested that, in Ang II-induced hypertensive chronic injured kidney, elevated endothelial HIF-1α contributes to the initial glomerular injury, leading to hypertension and progression of renal fibrosis [48]. However, other studies do not support a critical role of HIF-1 in glomerular endothelial patho-physiology. For example, Kalucka J et al. found that loss of HIF-1α in glomerular endothelial cells increases hypoxic cell death in vitro, but in vivo, HIF-1α expression in endothelial cells in mouse kidneys is detectable but limited. As a result, endothelial cell-specific ablation of HIF-1α does not have obvious effects on developmental phenotype in the kidney and also no influence of renal function and adhesion molecules expression during fibrosis development after UUO [49,53]. Kapitsinou P et al. further showed that inhibition of total endothelial HIF can aggravate renal fibrosis significantly in both ischemia-reperfusion and ureteral obstruction models. Specifically, inactivation of endothelial HIF-2α, but not HIF-1α, leads to increased expression of renal injury markers and fibrotic marker TGF-β1 in the post-ischemic kidney [49]. Furthermore, activation of HIF via PHD inhibition pharmacologically or genetically protects wild-type animals from ischemic kidney injury but the protective effects disappear in endothelial HIF-2α knockout animals, supporting a role of endothelial HIF-2α in the protective effects [49].

Moreover, HIF-1α has been reported to contribute to the profibrotic action of Ang II in renal medullary interstitial cells. During fibrosis, HIF-1α is induced along with fibrotic markers and transdifferention markers, including collagen I/III, the tissue inhibitor of metalloproteinase-1 (TIMP1), vimentin, and alpha-smooth muscle actin(α-SMA) [50]. Besides, both in UUO model and in kidney ischemia model, ablation of HIF-1α in podocytes is protective against glomerulosclerosis and glomerular type-I collagen accumulation in mice [51]. Apparently, more work is needed to understand the exact role of HIF in non-tubular epithelial cells in renal fibrosis and CKD.

## 4. Mechanisms of HIF Signaling in Renal Fibrosis

### 4.1. Transcriptional Regulation of Fibrogenic Genes

As a transcription factor, HIF can directly regulate the expression of fibrogenic factors by binding to the hypoxia responsive elements (HRE) in the promotor regions of these genes to activate gene transcription for the activation of fibroblasts and extracellular matrix remodeling and deposition (Figure 1). In anti-Tac(Fv)-PE38(LMB2)-induced podocyte injury and focal segmental glomerulosclerosis model, HIF-1α has been reported to increase and bind to the HRE of collagen type I alpha 2 chain (COL1A2) promotor. HIF-1α can form a transcription complex with smad family member 3(SMAD3) at this site, which is critical for the induction of collagen I after injury [51]. In addition, HIF-1 may also contribute to the transcriptional regulation of another important fibrogenic protein, plasminogen activator inhibitor-1 (PAI-1). PAI-1 can promote extracellular matrix deposition through inhibition of plasmin-dependent extracellular matrix degradation. In hypoxia-treated renal proximal tubular cells, the transcription of PAI-1 is strongly dependent on the induction and accumulation of nuclear HIF-1 [54]. The direct trans-activation of PAI-1 via HIF binding to the HRE in PAI-1 promoter region has also been reported during hypoxia of rat hepatocytes [55]. Furthermore, Norman JT et al. reported the direct transcriptional effect of HIF on tissue inhibitor of metalloproteinase 1 (TIMP-1). The induction of TIMP-1 in renal fibroblasts under hypoxia cannot be blocked by anti-TGF-β1 antibody and an HRE is also identified in TIMP-1 promotor [56]. In addition, it has been reported that HIF-1 induces gene transcription of collagen prolyl (P4HA1 and P4HA2) and lysyl (PLOD2) hydroxylases in fibroblasts, although it is unclear whether HIF can transactivate these fibrogenic genes in kidney. Thus, HIF-1 may also contribute to extracellular matrix remodeling in renal fibrosis by inducing the genes for collagen deposition, extracellular matrix stiffening, and collagen fiber alignment [57].

### 4.2. Crosstalk of HIF with Other Pro-Fibrotic Signaling Pathways

Besides the direct activating the transcription of fibrogenic factors, HIF can crosstalk with multiple pro-fibrotic signaling pathways, including TGF-β, Notch, NF-κB, and PI3K/Akt pathways, to further regulate renal fibrosis (Figure 1). TGF-β signaling pathway is a well-recognized pro-fibrotic pathway in CKD, and various studies have suggested a connection between HIF and TGF-β signaling pathways in renal fibrosis [58,59,60,61,62]. As mentioned above, HIF can enhance SMAD3-mediated transcription of COL1A2. In addition, HIF can directly activate the transcription of connective tissue growth factor (CTGF), a TGF-β signaling mediator) and synergistically induce renal fibrosis [62]. The modulation of TGF-β by HIF has also been demonstrated in an endothelial specific PHD2 knockout mouse model. When PHD2 is depleted, the accumulation of HIF will significantly up-regulate TGF-β1 expression in endothelial cells and promote renal fibrosis [63]. Conversely, TGF-β can also regulate the expression of HIF at transcription, translation, and degradation levels [58,59,60,61]. At transcriptional level, during TGF-β treatment of mesangial cells, mTORC1 and SMAD3 can interact with each other to enhance the expression of HIF-1 and the downstream collagen expression [59]. The translational level of HIF regulated by TGF-β1 is cell type specific. In renal tubular epithelial cells, more HIF-1α expression is stimulated by TGF-β1/SMAD3 signaling, while in mesangial cells, HIF-2α induction is more prevalent [58,61]. Even in normoxic condition, TGF-β1 treatment can induce HIF-1α in renal tubular cells. One potential mechanism is that TGF-β1 treatment and SMAD2/3 activation inhibit PHD2 expression resulting in HIF-1α accumulation [60].

Moreover, HIF can also interact with other signaling pathways in renal fibrosis regulation. In PHD2 knockout endothelial cells, HIF accumulation not only induces TGF-β1, but also significantly enhances Notch3 expression [63]. In addition, in angiotensin II infusion model of hypertensive chronic kidney disease, HIF-1α has been shown to be essential to initiate the glomerular injury and progression to renal fibrosis by transcriptional activation of genes encoding multiple vasoactive proteins. Mechanistically, the induction of endothelial HIF-1α gene expression is NFκB-dependent, and there is a reciprocal regulation between HIF-1α and NFκB [48]. In addition, HIF can interplay with PI3K/Akt to regulate fibrosis. HIF-1 induction in renal epithelial tubular cells can activate the transcription of Bmi-1, which will further modulate PI3K/Akt signaling and facilitate Snail-mediated EMT reaction to promote renal fibrosis [64]. Though not reported in kidney, in lung fibroblast, the activation of PI3K/Akt leads to transcriptional activation and protein expression of HIF-1α, which contributes to the fibroproliferative and collagen-inducing effects [65]. Other important signaling pathways, such as WNT/β-catenin and Hedgehog pathways, play important roles in renal fibrosis in CKD, but their crosstalk with HIF remains to be elucidated.

### 4.3. Epithelial-Meshenchymal Transition

EMT is defined as the epithelial cells undergoing the loss of cell-cell adhesion and apical-basal polarity, mesenchymal markers expression, rearrangement of the cytoskeleton, cell–cell dissociation, and obtaining a mesenchymal phenotype [66]. In CKD, the recent evidence implicates the absence of full EMT, which means that renal epithelial cells may not fully convert to fibroblast during injury [67]. However, partial EMT, where renal epithelial cells may change their phenotype to be fibroblast-like but still reside in tubules, may still have significant contribution to renal fibrosis [68,69,70,71]. Hypoxia or HIF-induced EMT is a well-known phenomenon in renal fibrosis in CKD conditions [45,60,64,72,73,74]. Under hypoxic circumstances, by direct transactivation or indirect regulation, HIF can activate several EMT transcriptional regulators such as Twist1, Snail, and Slug [64,74,75,76]. Specifically in kidney, HIF-1 in renal proximal tubular cells may promote EMT when activated by hypoxia or by overexpression in vitro [45,72]. In addition, selective knockout of HIF in mouse proximal tubular cells can remarkably reduce interstitial fibrosis, suggesting the possibility of communication between epithelial cells undergoing EMT and renal fibroblasts [45]. Several potential underlying mechanisms have been reported for HIF to regulate EMT in CKD condition, which include the induction of Twist, activation of lysyl oxidase, and the up-regulation of Bmi1 and the stabilization of Snail through PI3K/Akt pathway [45,64,74].

### 4.4. Epigenetic Regulation

Epigenetics is a mechanism of regulating gene transcription by direct chemical modification of DNA and by modification of proteins that are closely associated with the locus without changing of DNA sequence. Common forms of epigenetic regulation include DNA methylation, histone modification, chromosome conformation, microRNA, and long non-coding RNAs. In CKD, DNA methylation is highly induced, which has been shown to facilitate the development of renal fibrosis [77,78]. Meanwhile, histone methylation change has been reported in mesangial cells and epithelial cells in diabetic condition [79,80]. In addition, histone acetylation and histone ubiquitination are also up-regulated in fibrotic kidney [81,82]. In addition, in aging nephropathy, chromatin conformation change contributes to the induction of collagen III with more recruitment of RNA polymerase II for gene transcription [83]. Moreover, multiple microRNAs and long non-coding RNAs have been implicated to promote renal fibrosis development in various CKD conditions. All the evidence indicates the critical role of epigenetic regulation in the pathogenesis of CKD.

Even though there is intense research interest in the epigenetic regulation in renal fibrosis and hypoxia is also a well-known condition in CKD, the involvement of HIF in epigenetic regulation under CKD condition has not been well studied. Nevertheless, hypoxia has been reported to reduce CTGF expression (an ECM stimulator) in human kidney epithelial cells with the involvement of DNA methylation induction [84]. HIF also functions on the regulation of both pro-fibrotic microRNA such as microRNA-155(miR-155) [85], and anti-fibrotic microRNAs such as miR-29 [41]. However, the evidence of whether HIF can modulate histone modification, chromatin change or long non-coding RNA expression in kidney is still lacking and the detailed underlying mechanism for HIF to regulate renal cell epigenetic change in CKD remains unclear.

## 5. HIF in CKD-Associated Syndromes

### 5.1. Anemia

EPO plays an essential role in the proliferation and differentiation of erythroid progenitors and regulation of tissue oxygen supply [86]. The adult kidney, as the main source of EPO production, is the critical organ for the regulation of erythropoiesis. Therefore, for CKD patients, with the progression of chronic kidney injury and renal function loss, anemia is a common complication predominantly resulting from the deficiency of renal EPO production [87,88]. EPO is mainly synthesized in fibroblast-like interstitial cells located in kidney [89,90,91]. The production of EPO is regulated in a hypoxia-inducible manner [32]. HIF, as the main transcriptional regulator in hypoxia, was initially identified to bind to human erythropoietin Gene Enhancer which is required for transcriptional activation of EPO [92]. The HIF signaling pathway can modulate and coordinate erythropoiesis at multiple levels including stimulating EPO production, promoting the uptake and utilization of iron, and altering the bone marrow microenvironment to facilitate erythroid progenitor maturation and proliferation [88,93]. HIF-2 is the key regulator of hypoxic EPO induction and is required for normal erythropoiesis [94], and HIF-2 deletion from renal tissue results in severe anemia [95]. In contrast, the role of HIF-1 in EPO production is unclear and may depend on cell types and context. PHD2 inactivation-induced HIF-2 stabilization leads to EPO overproduction, which is further increased by co-inactivation of HIF-1 possibly because HIF-1 can promote PHD3 expression to neutralize the effect of PHD2 [96]. However, there is also report about HIF-1-mediated EPO production. Herpesvirus entry mediator (HVEM) in renal macrophages was reported to promote NO production and HIF-1α activation in kidney that further induced EPO [97]. In CKD, both HIF-1 and HIF-2 are activated, rendering it hard to determine their effects on anemia [96]. Interestingly, even though EPO is mainly produced by interstitial cells, HIFs in epithelial cells may play a regulatory role. HIF activation in kidney proximal tubules via VHL-knockout led to a decrease in EPO-producing interstitial cells and the development of hypoproliferative anemia in mice, suggesting HIF-mediated crosstalk between epithelial cells and interstitial cells [98].

The control of EPO synthesis by HIF-2 in kidney is prominently regulated by PHD1/2/3 which function as cellular oxygen sensors [99,100,101,102,103]. Kobayashi H et al. have reported that EPO-producing cells are entirely derived from FOXD1-expressing stromal progenitor cells and identified PHD2 as the main regulator with adjunctive regulation from PHD1 and PHD3. They showed that PHD2 inactivation alone induced renal EPO in a limited number of renal interstitial cells and hypoxia or pharmacologic PHD inhibition further increased the renal EPO producing cell fraction among PHD2^−/−^ renal interstitial cells. Moreover, heterozygous deficiency for PHD1 and PHD3 further increased renal EPO producing cell numbers in PHD2^−/−^mice. Therefore, PHD2 mainly regulates the EPO producing capacity while PHD1 and PHD3 may modulate the number of renal EPO producing cells. Because FOXD1 lineage renal interstitial cells consist of distinct subpopulations, the heterogeneity of EPO-producing renal interstitial cells may cause variations of PHD activity levels , their ability to regulate HIF-2α degradation, and EPO production under hypoxia conditions [104].

In addition to the direct regulation by PHDs, HIF-2 stabilization for EPO production is also under post-translational regulation. In hypoxia, HIF-2α is rapidly acetylated by lysine acetyltransferase cAMP-response element binding protein (CREB)-binding protein which is further controlled by acetate-dependent acetyl CoA synthetase 2 [100,105]. This acetylation promotes HIF-2 mediated EPO production. Soon after the acetylation, Sirt1 will be activated to selectively deacetylate the lysine at C-terminal of HIF-2α, which will augment HIF-2α transcriptional activity [101].

In 2011, Yanagita and colleagues demonstrated that there is a specific population of interstitial fibroblasts in kidneys that produce EPO and, during CKD development, these EPO-producing cells (REPs) may transdifferentiate into myofibroblasts resulting in the loss of their EPO-producing ability and anemia [106]. By live imaging, Souma T et al. [93] recently showed that healthy REPs associate with endothelium by wrapping processes around capillaries tightly, and this association is blocked by inflammation in renal injury resulting in the transition of REPs to myofibroblast-transformed renal EPO-producing cells (MF-REPs). Activation of HIFs may reactivate EPO production in MF-REPs [93]. Moreover, in CKD condition, when REPs differentiate into myofibroblast, TGF-β1 signaling may cause the hypermethylation of EPO gene, which reduces HIF-2 related EPO transactivation. Thus, anemia can be ameliorated by 5-azacytidine treatment to inhibit DNA methyltransferases [107].

### 5.2. Inflammation

Hypoxia and inflammation frequently coexist and have been shown to modulate each other. Hypoxia can affect inflammatory cell recruitment, and inflammatory factor production and secretion. Conversely, inflammatory cells modulate the activation of hypoxic signaling pathways [108,109,110]. The vital role of HIF in kidney inflammation has been shown in various kidney disease models including CKD [18,49]. Inactivation of HIF-1 signaling in renal epithelial cells is associated with decreased inflammatory cell infiltration in CKD and renal fibrosis condition [45]. In the remnant kidney and Thy1 nephritis models, HIF activated by cobalt chloride reduces macrophage infiltration [111,112]. In mouse UUO model, global or conditional knockout of HIF in myeloid cells show more severe inflammation, while HIF activation by myeloid-specific VHL-knockout only suppresses inflammation but without obvious effect on renal fibrosis [42]. Although both HIF-1 and HIF-2 activation contribute to inflammatory response, they have distinct roles in macrophage phenotype, as HIF-1 is more related to M1 and HIF-2 to M2 [42]. Moreover, it is still important to maintain the physiological low level of HIF in normal conditions. When VHL is selectively knocked out in collecting ducts and a subset of distal tubules, due to the over accumulation of HIF, the mice exhibit wide-spread epithelial disruption and interstitial inflammation as early as 2 months of age with abundant infiltrating macrophages and lymphocytes. The VHL mutant lesions can be rescued by HIF-1α knockout [22].

In endothelial cells, HIF-1 and HIF-2 may play different roles in inflammation in CKD. According to Kalucka J et al., loss of HIF-1α in glomerular endothelial cells reduces hypoxic adhesion of macrophages in vitro. However, in vivo work indicates that HIF-1α expression in endothelial cells in mouse kidneys is detectable but limited. Therefore, endothelial cell-specific ablation of HIF-1α has no effect on kidney development and no influence on renal function and adhesion molecules expression during inflammation after UUO [53]. The limited role of endothelial HIF-1 in inflammation is also confirmed in another study [49]. In both ischemia-reperfusion and ureteral obstruction induced renal fibrosis models, inactivation of endothelial HIF-2α, instead of HIF-1α, can result in enhanced inflammatory cell infiltration. Pharmacologic or genetic activation of HIF via PHD inhibition protects against ischemic kidney injury and inflammation in wild-type animals, but not in animals lacking endothelial HIF-2 [49]. Therefore, in endothelial cells, HIF-2, rather than HIF1, plays a major role in regulating the inflammatory responses in CKD. 

Meanwhile, HIF signaling may also be regulated by inflammatory response. In a recent study, Yamaguchi J et al. identified CCAAT/enhancer-binding protein δ (CEBPD) as a new HIF-1 regulator [18]. CEBPD is a transcription factor originally identified as an inflammatory response gene [113,114]. It is up-regulated in proximal tubular cells in multiple AKI and CKD models including hypoxia injury and the induction of CEBPD further promotes the transcription of HIF-1α [18]. Currently, it is still obscure whether HIF-2 signaling can also be regulated by inflammatory response and more evidence is still needed to elucidate the feedback of inflammation to hypoxia.

### 5.3. Aberrant Angiogenesis

The complicated renal vascular system governs the balance maintenance between oxygen consumption and supply. The progression of CKD is associated with renal capillary rarefaction and abnormal angiogenesis, leading to persistent capillary loss and hypoxia in renal tissues [7]. HIF, as the key transcriptional regulator in hypoxia, is activated in this process resulting in the induction of various angiogenic factors for angiogenesis [115,116]. Selective deletion of VHL in renal epithelium in mice to stabilize HIF causes increased medullary vascularization and this phenotype is completely rescued by HIF-1α co-deletion but not by HIF-2α co-deletion [117]. Among all uncovered angiogenic factors, vascular endothelial growth factor (VEGF) is critical in the maintenance of peritubular capillaries. As the direct target gene of HIF, VEGF is primarily controlled by HIF-1 [118,119]. However, excessive VEGF expression in the tubular cells may be detrimental, because increased tubular VEGF expression may propel fibrosis and cyst formation, and aggravate kidney injury [120,121]. Though direct evidence of HIF-2 regulated renal angiogenesis is lacking, Skuli N et al. examined the detailed mechanism of HIF-2 mediated vascular function and angiogenesis in muscle ischemic model [122]. Using endothelial cell HIF-2α specifically deleted mice, they found that HIF-2α deletion resulted in increased vessel formation and improper arteriogenesis. HIF-2α deficient endothelial cells have increased migration, invasion, and morphogenetic activity, which are mediated by specific angiogenic factors, including δ-like ligand 4 (Dll4), a Notch ligand, and angiopoietin 2 [122]. Collectively, these data indicate that HIF-1 and HIF-2 may have complementary effects on vascular remodeling and angiogenesis [122].

### 5.4. Vascular Calcification

Cardiovascular complications are known to be the leading cause of death in patients with CKD and vascular calcification is a common complication in CKD. The extent and histoanatomic type of vascular calcification are effective predictors of subsequent vascular mortality in CKD patients [123]. During CKD, multiple factors, such as oxidative stress, dyslipidemia, advanced glycation end products, and disordered mineral metabolism, may contribute to vascular calcification [124]. All these injury factors dramatically increase especially when patients start dialysis [125]. During CKD, due to metabolic disorders, the vascular smooth muscle cells may transform to a “osteoblast-like” phenotype, causing calcium phosphate deposition in the extracellular matrix [126].

HIF-1 has been reported to play a crucial role in vascular smooth muscle cell (VSMC) calcification [127]. During the progression of CKD, VSMC calcification and osteogenic trans-differentiation are significantly promoted by hypoxic condition. HIF-1 depletion in VSMC can block the calcification under hypoxia and elevated inorganic phosphate condition. Meanwhile, HIF-1 activators can further promote inorganic phosphate induced calcification. Together, hypoxia can synergize with elevated inorganic phosphate through HIF-1 induction to enhance VSMC osteogenic transdifferentiation [127]. Furthermore, Bone Gla Protein (BGP), a marker for bone formation which is induced in calcified vasculatures, may also promote HIF-1α expression and further stimulate the calcification [128]. In addition, clinical data also implicate close relationship between HIF and vascular calcification. Both HIF-1α and HIF-2α activation have been localized in calcified aorta valve [129,130]. Li G et al. also showed that elevated serum HIF-1α may be involved in coronary artery calcification by analysis of the computed tomography scanning data of 405 patients with type 2 diabetes mellitus [131]. Altogether, these studies support an indispensable role of HIF in vascular calcification, making it a potential therapeutic target.

## 6. Therapeutic Outlook

Given the evidence for an essential role of hypoxia and HIF in CKD initiation, progression and related complications, HIF and related pathways may be therapeutic targets in CKD-related pathological conditions. The use of inhibitors or stabilizers to target HIF signaling pathway for the therapy of CKD and related complications are under intensive investigation. Some of them have already proceeded into clinical application and more are in clinical trials [132,133,134,135,136].

So far, most researches of HIF signaling pathway targeted treatments are focusing on anemia because they are more physiologic relevant than the conventional erythropoiesis-stimulating agents (ESAs) [88,137]. HIFs coordinate a series of biological processes to induce EPO and erythropoiesis. Several recent clinical studies have demonstrated the beneficial effects of PDH inhibitors in treating anemia in CKD patients [132,133,134,135,136,138]. By blocking PHDs, these reagents induce the stabilization and accumulation of HIFs to stimulate EPO production and effective erythropoiesis to reduce anemia in CKD [139,140]. One advantage of PHD inhibitors (or HIF stabilizers) comparing to traditional treatments is that these agents are administered orally, which is remarkably helpful for both patients not entering dialysis and those under peritoneal dialysis [141]. Furthermore, HIF stabilizers induce physiological EPO production, not like ESA therapies that may cause abnormal and harmful high peak EPO [142].

Although the application of HIF-targeted therapies has led to notable advances in treating anemia of CKD, it also has some disadvantages or limitations. First, HIF and hypoxia are involved in the regulation of diverse biological procedures. Persistent HIF activation over prolonged periods may cause extra side effects, including changes in glucose levels, fat and cholesterol metabolism, and the possible promotion of tumor growth [141]. Furthermore, because HIF have different pathological roles in distinct renal cells in CKD, it is a challenge to prevent the potential adverse effect on the development of renal fibrosis, angiogenesis, and vascular calcification by global activation of HIF. Therefore, despite the promising outcome of targeting HIF therapy in certain CKD and related complications, further research needs to delineate the possible adverse effects and design more specific therapeutic strategies.
